# Rapid recovery of tropical forest diversity and structure after shifting cultivation in the Philippines uplands

**DOI:** 10.1002/ece3.6419

**Published:** 2020-06-05

**Authors:** Sharif A. Mukul, John Herbohn, Jennifer Firn

**Affiliations:** ^1^ Tropical Forests and People Research Centre University of the Sunshine Coast Maroochydore QLD Australia; ^2^ Tropical Forestry Group School of Agriculture and Food Sciences The University of Queensland Brisbane QLD Australia; ^3^ School of Earth, Environmental and Biological Sciences Faculty of Science and Engineering Queensland University of Technology Brisbane QLD Australia

**Keywords:** *kaingin*, reforestation, restoration, secondary forest, Southeast Asia, succession, tree diversity

## Abstract

Shifting cultivation is a widespread land‐use in the tropics that is considered a major threat to rainforest diversity and structure. In the Philippines, a country with rich biodiversity and high rates of species endemism, shifting cultivation, locally termed as kaingin, is a major land‐use and has been for centuries. Despite the potential impact of shifting cultivation on forests and its importance to many people, it is not clear how biodiversity and forest structure recover after kaingin abandonment in the country, and how well these post‐kaingin secondary forests can complement the old‐growth forests. We investigated parameters of forest diversity and structure along a fallow age gradient in secondary forests regenerating after kaingin abandonment in Leyte Island, the Philippines (elevation range: 445–650 m asl). We first measured the tree diversity and forest structure indices in regenerating secondary forests and old‐growth forest. We then measured the recovery of tree diversity and forest structure parameters in relation to the old‐growth forest. Finally, using linear mixed effect models (LMM), we assessed the effect of different environmental variables on the recovery of forest diversity and structure. We found significantly higher species density in the oldest fallow sites, while Shannon’s index, species evenness, stem number, basal area, and leaf area index were higher in the old‐growth forest. A homogeneous species composition was found across the sites of older fallow age. Multivariate analysis revealed patch size as a strong predictor of tree diversity and forest structure recovery after shifting cultivation. Our study suggests that, secondary forests regenerating after shifting cultivation abandonment can recover rapidly. Although recovery of forest structure was not as rapid as the tree diversity, our older fallow sites contained a similar number of species as the old‐growth forest. Many of these species are also endemic to the Philippines. Novel and emerging ecosystems like tropical secondary forests are of high conservation importance and can act as a refuge for dwindling tropical forest biodiversity.

## Graphical abstract

We investigated parameters of forest diversity and structure along a shifting cultivation fallow gradient in Leyte island, the Philippines. Regenerating secondary forests following shifting cultivation abandonment can exhibit high resilience and recover rapidly. Novel and emerging ecosystems like tropical secondary forests are of high conservation importance and can act as a refuge for dwindling tropical forest diversity.

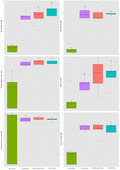

## 1 INTRODUCTION

Large areas of tropical forest have been modified by human activity, mainly logging and shifting cultivation, and the persistence of forest in the tropics relies on the effective management of such human‐modified landscapes (Chazdon, 2008, 2014; Gardner et al., 2009). In recent years, secondary or second‐growth forests in the tropics have become a key concern for scientists and policy makers due to their recognized potential to offset the unprecedented loss of tropical forest biodiversity (Chazdon et al., 2009; Laurance, 2007; Pichancourt, Firn, Chades, & Martin, 2014). It is also increasingly acknowledged that regenerating secondary forests in the tropics can provide environmental benefits equivalent to primary forests, although the role they have played in biodiversity conservation just recently begun to understand (see—Mukul, Herbohn, & Firn, 2016a; Bonner, Schmidt, & Shoo, 2013; van Breugel et al., 2013; Chazdon et al., 2009; Norden, Chazdon, Chao, Jiang, & Vilchez‐Alvarado, 2009; Rozendaal et al., 2019).

In Southeast Asia, shifting cultivation or slash‐and‐burn agriculture has been considered a primary agent of forest loss and degradation (Geist & Lambin, 2002; Houghton, 2012; Ziegler et al., 2012). Shifting cultivation is a dominant land use in this region with deforestation rates still remaining high (Dalle, Pulido, & de Blois, 2011; Sodhi et al., 2010). In recent years, although the extent of land under shifting cultivation has declined in many countries (van Vliet et al., 2012), this traditional land use still forms a central part of the livelihoods of millions of upland rural smallholders in Southeast Asia (Dalle et al., 2011; Mertz et al., 2009). At the same time, significant debate persists among policy makers and the scientific community over the impact of shifting cultivation on forest dynamics from both a management and conservation perspective (Mukul & Herbohn, 2016; van Vliet et al., 2012).

Negative perceptions of shifting cultivation's impacts on forest diversity and structure mainly center around comparisons with primary or less‐disturbed forests with yet very limited studies from Southeast Asia (see—Castro‐Luna, Castillo‐Campos, & Sosa, 2011; Ding, Zang, Liu, He, & Letcher, 2012; Do et al., 2011; Klanderud et al., 2010). Due to the dynamic nature of the landscapes, including differences in site management and spatial heterogeneity, research into the impacts of shifting cultivation on secondary forest dynamics has always been challenging (Mukul & Herbohn, 2016). In the tropics, the intensity of shifting cultivation and past land use is an important factor in determining the capacity of regenerating forests to recover in terms of species diversity, composition, and forest structure, although a large variation exists in the recovery of different forest attributes and the time required for recovery (Jakovac, Peña‐Claros, Kuyper, & Bongers, 2015; Lawrence, 2004; Lawrence, Radel, Tully, Scmook, & Schneider, 2010; Villa, Martins, Oliveira Neto, Rodrigues, Martorano, et al., 2018; Villa, Martins, Oliveira Neto, Rodrigues, Vieira, et al., 2018). For example, some research suggests it can take at least ten years for woody species to become prominent in secondary forests following shifting cultivation (Raharimalala et al., 2010) and 60 years to achieve the biodiversity levels of an old‐growth forest (Do et al., 2011). Others have reported the recovery of specific stand structure indices, such as stand density, can be rapid in young fallow areas (McNamara, Erskine, Lamb, Chantalangsy, & Boyle, 2012), although the recovery of other forest structure parameters may take as long as 40 years (Piotto, Montagnini, Thomas, Ashton, & Oliver, 2009). In many cases, an active forest management strategy has been found to support a more rapid restoration of tree diversity and forest structure (Bonilla‐Moheno & Hol, 2010).

The Philippines maintain about 5% of the world's plant diversity with a high level of species endemism (Lasco, Veridiano, Habito, & Pulhin, 2013; Sodhi et al., 2010). The forest cover in the country declined from 50% in 1950s to 24% in 2004 (Lasco et al., 2013), with the majority of the remaining forests being severely degraded (Chokkalingam et al., 2006). Shifting cultivation, locally known as *kaingin,* is a common, yet controversial land use in the country (Saurez & Sajise, 2010). *Kaingin* has been blamed for much of the country's deforestation and forest degradation, and it is not a recognized land use in government policies (Kummer, 1992; Lasco, Visco, & Pulhin, 2001). After post‐logging secondary forest, post‐*kaingin* secondary forest represents the second largest group of secondary forests in the Philippines (Lasco et al., 2001). In the Philippine, as in other Southeast Asian countries, secondary forests regenerating after shifting cultivation abandonment are becoming more common (Chokkalingam & Perera, 2001). At the same time, it is not clear how well these post‐*kaingin* secondary forests can complement the old‐growth forests, and how biodiversity and forest structure recover after *kaingin* abandonment in the country.

We investigated the recovery of tree species diversity and forest structure along a fallow gradient in secondary forests regenerating after shifting cultivation abandonment in an upland area of the Philippines—a global biodiversity hotspot and a megadiverse country (Posa, Diesmos, Sodhi, & Brooks, 2008). Species diversity was measured in terms of tree species density, Shannon–Wiener index, and species evenness index, while forest structure was measured in terms of stem density, basal area, and leaf area index (LAI). We also examined the factors that may expedite the recovery process in regenerating secondary forest ecosystems. We believe our study is useful to not only recognize the recovery potential of regenerating forests after shifting cultivation abandonment but also to better understand the prospect of such landscapes to support forest restoration and conservation in the Philippines and other Southeast Asian countries.

## 2 MATERIALS AND METHODS

### 2.1 Study area

**FIGURE 1:**
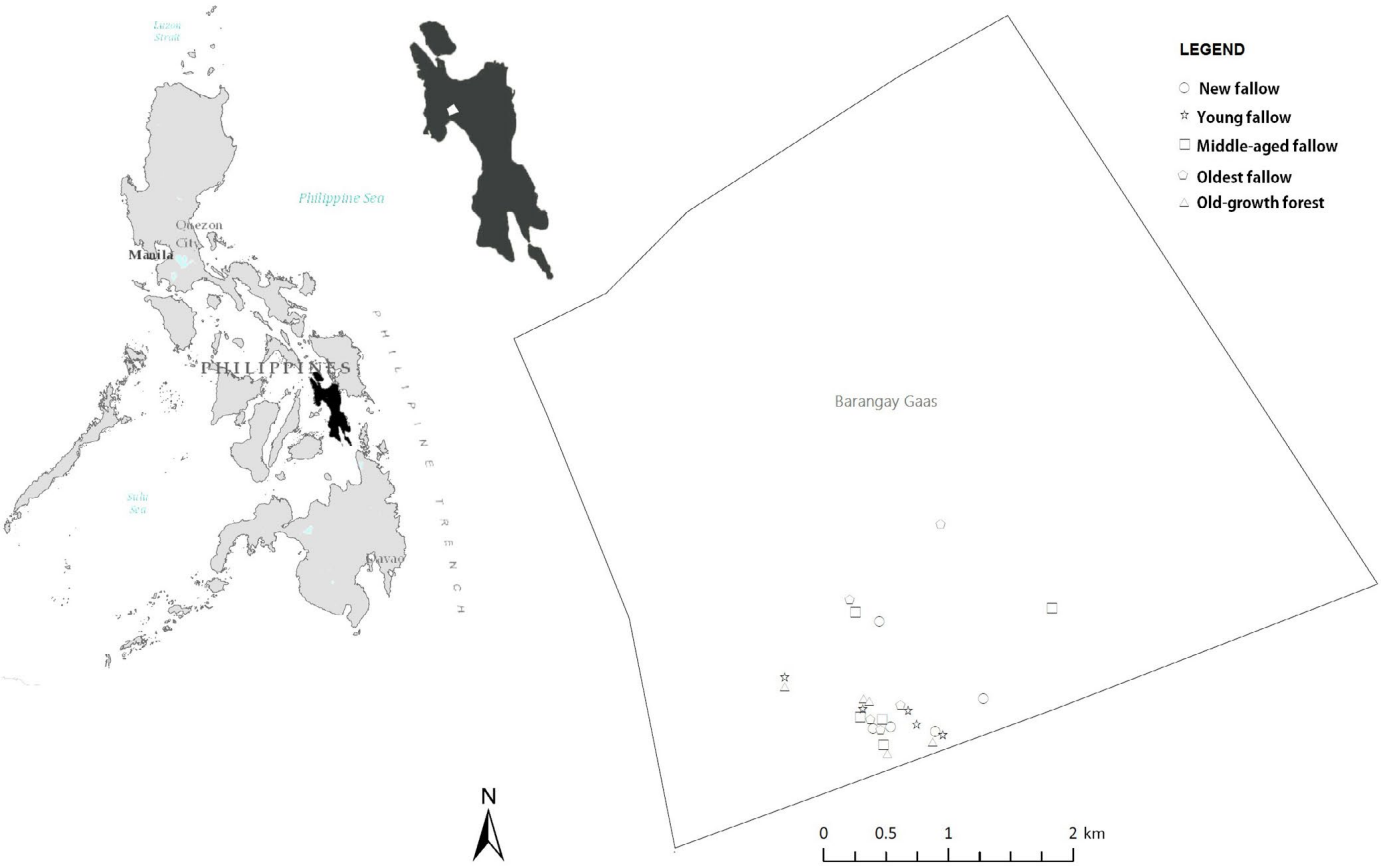
Map showing the location of our study area on Leyte Island and the study sites in Gaas

We conducted our study in Barangay (the smallest administrative unit in the Philippines and the native Filipino term for a village, Brgy. in short) Gaas in Ormoc city—located in the west part of Leyte Island in the Philippines. Leyte is the eighth largest island in the country with an area of about 800,000 ha. Geographically, it is situated between 124°17' and 125°18^'^ East longitude and between 9°55' and 11°48' North latitude (Figure 1). Forest cover on the island is about 10%, although the once dipterocarp‐rich rainforests of the island are now represented by patches of old‐growth and secondary forests intermixed with coconut (*Cocos nucifera*) and abaca (*Musa textilis*) plantations (Asio, Jahn, Stahr, & Margraf, 1998). The relatively flat lowlands are used for agricultural production, mainly rice, corn, and sweet potatoes (Asio et al., 1998). There have been many attempts to support reforestation on Leyte Island, and there are numerous smallholder and community forests scattered across the island, including “rainforestation plantings” which were designed to reduce *kaingin* activity (Nguyen, Lamb, Herbohn, & Firn, 2014).

Leyte has a “type IV” climate based on the Coronas Classification of Climate (Mukul, 2016). The island enjoys a relatively even distribution of rainfall throughout the year with an annual rainfall of about 4,000 mm (Jahn & Asio, 2001). Although there is no distinct dry season, between March and May the area experiences its lowest rainfall. Mean monthly temperature is 28°C which remains relatively constant throughout the year (Navarrete, Tsutsuki, & Asio, 2013). Relative humidity ranges between 75% and 80% during the dry and the wettest months (Jahn & Asio, 2001).

### 2.2 Site selection criteria

Olofson (1980) categorized the *kaingin* systems in the Philippines into three distinct types based on the sites where they have been practiced. These are as follows: (a) the *tubigan* system; (b) the *katihan* system; and (c) the *dahilig* system. The *tubigan* system involves the use of irrigation water and work animals in lowland areas, the *katihan* system is common in gently sloping land with limited facilities for irrigation, whereas the *dahilig* system is widely practiced on steeper slopes (Olofson, 1980). For the present study, we sampled only the *dahilig* system as it is comparable to the most common form of shifting cultivation found in much of Southeast Asia.

We purposively chose Brgy. Gaas (hereafter Gaas only) for our study. It is situated in an area of relatively high elevation (between 445 and 650 m asl) with a low population density and high forest cover, two essential conditions for the successful regeneration of *kaingin* fallow secondary forests (Chokkalingam et al., 2006). Smallholders living in the area usually grow abaca or coconut in their *kaingin* fallow land in order to receive some cash benefits during the time of abandonment. Our study was, however, confined to the areas where farmers planted only abaca since coconut plantations generally lead to a more intensive land management and as such secondary forest regrowth generally does not result.

### 2.3 Sampling protocol and vegetation survey

Chronosequence studies (a time for space replacement) are popular as an alternative to long‐term ecological research and have been widely used to investigate successional trends in tropical forests (Johnson & Miyanishi, 2008; Norden et al., 2015; Pickett, 1989). We categorized our secondary forest sites into four different fallow age categories; that is, fallow less than (or equal to) 5 years old, hereafter referred to as new; 6‐ to 10‐year‐old fallow, hereafter referred to as young; 11‐ to 20‐year‐old fallow, hereafter referred to as middle‐aged; and 21‐ to 30‐year‐old fallow, hereafter referred to as oldest fallow. Additionally, old‐growth forest sites without any history of *kaingin* and logging and located close to the fallow sites were sampled as the control. Our control old‐growth forest sites were indispensable references against which we compare fallow sites in order to properly assess the forest recovery and resilience (Norden et al., 2009).

Vegetation surveys in our 25 sites (4 fallow age category + old‐growth forest × 5 replicates; total sample area 2.5 ha) were undertaken from May to October 2013. In the secondary forests, we sampled from the sites that were at least 1 ha in size (Piotto et al., 2009). Four transects of 50 m × 5 m (parallel to each other and a minimum of 5 m distance from each other) were established at each of our sites. We recorded diameter of each tree ≥5 cm at diameter at breast height (dbh) using a diameter tape.

Where possible trees were identified to the species level with the help of a local expert from Visayas State University (VSU). In the case of unknown species, we used the most common Filipino name of that species. Additional information, like global (as per IUCN, 2014) and local (as per DENR, 2007) conservation status, biogeographic origin, and successional guild were also collected.

### 2.4 Site environmental parameters

Both fallow age and the number of fallow cycles are known to influence vegetation and soil parameters and their recovery (Klanderud et al., 2010; Lawrence, Suma, & Mogea, 2005). Here, we are only able to consider fallow age and not the number of fallow cycles because reliable information was not available from the smallholders. Other site attributes collected were—elevation, patch size, slope, LAI, distance from the nearest old‐growth forest, and soil organic carbon (SOC %) (Table 1). We used a digital plant canopy imager (Model: CID Bio‐Science) for LAI and a hand‐held GPS (Model: Garmin eTrex) for site elevation.

**TABLE 1:** Environmental attributes of our fallow sites and old‐growth forest on Leyte Island, the Philippines

Site attributes	Fallow age category				Old‐growth forest	*F‐*value^a^
	New	Young	Middle‐aged	Oldest		
Elevation (m asl)	600.8 ± 22.19	549.0 ± 72.41	567.2 ± 49.24	574.8 ± 35.35	512.4 ± 54.77	2.18
Slope (degree)	33 ± 5.7	32.4 ± 9.4	32.6 ± 9.2	38.2 ± 7.98	36.4 ± 9.71	0.47
Patch area (ha)	1.16 ± 0.21	1.14 ± 0.13	1.34 ± 0.24	1.14 ± 0.22	na	—
Distance (m)	290 ± 74.16	540 ± 114.01	162 ± 198.17	256 ± 153.88	0	11.97^a^
SOCǂ (%)	6.17 ± 0.68	6.54 ± 2.05	5.21 ± 0.69	6.79 ± 1.92	4.77 ± 1.11	1.87

^a^ *F*‐value significant at *p* < .01 level as indicated from ANOVA.

### 2.5 Diversity and forest structure indices

Species diversity was described in terms of species density (S), Shannon–Wiener's diversity index (H) and species evenness index (J). Shannon's diversity index and species evenness index were calculated as described in Magurran (2004) while species density was the number of unique tree species per site. We used stem density (N), basal area (BA)*,* and LAI as the measure of forest structure. Both stem density and basal area (m^2^) were expressed on a per site (0.1 ha) basis. Stem density or number was the number of tree individuals (≥5 cm dbh) per site while basal area (m^2^) was the total cross‐sectional area of all stems (≥5 cm dbh) in each site. LAI was measured as the ratio between total leaf area and ground area.

### 2.6 Species composition and similarities

We used importance value index (IVI) to compare the patterns of tree species dominance in each of the secondary forest of different fallow age categories and in our control old‐growth forest. IVI was the sum of relative density, relative dominance, and relative frequency of species (Magurran, 2004). Permutational multivariate analysis of variance (PERMANOVA) was also performed to test whether species composition differed among different site categories, and a nonmetric multi‐dimensional scaling (NMDS) to assess species compositional similarities between secondary forest of different fallow age and control old‐growth forest.

### 2.7 Recovery of tree species diversity and forest structure

Recovery of tree diversity and forest structure were compared against the control old‐growth forest. We calculated recovery as the percentage of tree diversity and forest structure compared to our control forest sites using the following equation.

$$R=\frac{X_{\text{fallow}}}{X_{s}}\times 100,$$

where *X*_fallow_ is the measure of a diversity and/or forest structure parameter of the fallow site, and *X*_s_ is the mean of corresponding biodiversity and/or forest structure parameter in the control forest sites.

### 2.8 Data analysis

Statistical analysis was performed using the R package (version 3.0.1; R Development Core Team, 2014). Analysis of variance (ANOVA) and Tukey post hoc test were performed to test for significant differences between the variables. We used “Biodiversity R” (version 2.4‐1; Kindt & Coe, 2005) and “vegan” (version 2.0‐10; Oksanen et al., 2010) for calculating species richness and other community diversity indices. For PERMANOVA and NMDS, we used the Bray–Curtis similarity metric with 1,000 iterations, starting with a random configuration using the “vegan” package (Oksanen et al., 2010).

Linear mixed effect models (hereafter referred to as LMM) were developed to examine the effect of fallow age and site environmental attributes on tree diversity and forest structure recovery, using the package “nlme” (Pinheiro, Bates, Roy, & Sarkar, 2011). In our LMM, fallow age (FA), slope (SL), distance from nearest old‐growth forest (DIS), patch size (PS), and soil organic carbon (SOC) were used as explanatory variables (i.e., fixed factors), and site diversity (i.e., species density, Shannon's index, Species evenness) and forest structure indices (i.e., stem density, basal area, LAI) were the response variables. We used sites nested in fallow categories as the random effect in our models. Due to its collinearity with other explanatory variables, “elevation” was excluded from the final LMM (Appendix 1). We considered Akaike information criterion (AICc) corrected for small sample sizes for the selection of our top models, where the best models had the lowest AICc scores (Johnson & Omland, 2004). For our model selection and to evaluate the contribution different fixed effects had on explaining the variation in the response variables, we used the “MuMin” package in R (Bartoń, 2011). Models within four AICc units were considered equally supported among competing models (Grueber, Nakagawa, Laws, & Jamieson, 2011).

## 3 RESULTS

### 3.1 Species and characteristics

Altogether, we censused 2,918 tree individuals belonging to 131 species, 86 genera, and 46 families with six species remaining unidentified (see Appendix 2). There were no standing trees in two of our new fallow sites. Among the species, 14 belonged to the family Moraceae, followed by Dipterocarpaceae (10 species), Phyllanthaceae (8 species), Fabaceae (6 species), Euphorbiaceae (5 species), Lamiaceae (5 species), and Rubiaceae (5 species). At the landscape level, 106 species were recorded alone from our oldest fallow sites, followed by our middle‐aged fallow sites (95 species), and 79 species from the old‐growth forest. We found 40 species that were endemic to the Philippines. The highest number of endemic species was recorded in the oldest fallow sites (37 species), followed by the middle‐aged sites (30 species), the young fallow sites (29 species), and the old‐growth forest (27 species). Six exotic species were also recorded from the sites, although there were no exotic species in the old‐growth forest or in the young fallow sites. The highest number of pioneer (49), secondary (46), and climax (11) species were recorded in the oldest fallow sites (Table 2). We found nine tree species that are critically endangered globally (according to the IUCN Red List) in our oldest fallow sites, and eight species in both the young fallow sites and the middle‐aged fallow sites (Table 2; Appendix 2).

**TABLE 2:** Distribution and characteristics of species recorded from secondary forest and old‐growth forest sites on Leyte Island, the Philippines

Parameter	Fallow age category				Old‐growth forest
	New	Young	Middle‐aged	Oldest	
Biogeographic origin					
Endemic	4 (±1.10)	21 (±9.36)	20 (±13.16)	27 (±7.35)	21 (±7.31)
Native	23 (±12.32)	63 (±24.12)	71 (±29.34)	75 (±9.04)	58 (±13.44)
Exotic	1 (±0.45)	0	4 (±3.83)	4 (±3.11)	0
Successional guild					
Pioneer	12 (±3.44)	36 (±1.82)	47 (±3.42)	49 (±3.9)	35 (±3.83)
Secondary	12 (±3.71)	38 (±3.54)	40 (±4.92)	46 (±3.54)	35 (±1.64)
Climax	4 (±0.84)	10 (±1.79)	8 (±2.19)	11 (±1.52)	9 (±1.48)
Conservation status (Global)					
Critically endangered	3 (±0.89)	8 (±0.71)	8 (±2.07)	9 (±1.82)	6 (±1.22)
Endangered	0	0	1 (±0.45)	0	0
Vulnerable	2 (±0.89)	9 (±2.07)	10 (±2.68)	10 (±1.3)	11 (±1.34)
Conservation status (Local)					
Critically endangered	0	3 (±0.84)	3 (±0.55)	3 (±0.84)	3 (±0.71)
Endangered	1 (±0.55)	2 (±0.55)	2 (±0.55)	1 (±0.55)	1 (±0.55)
Vulnerable	2 (±0.45)	5 (±0.89)	5 (±1.52)	6 (±1.0)	5 (±0.45)

Values in the parenthesis indicates the standard deviation (*SD*) of mean.

### 3.2 Biodiversity and forest structure in regenerating secondary forests

**FIGURE 2:**
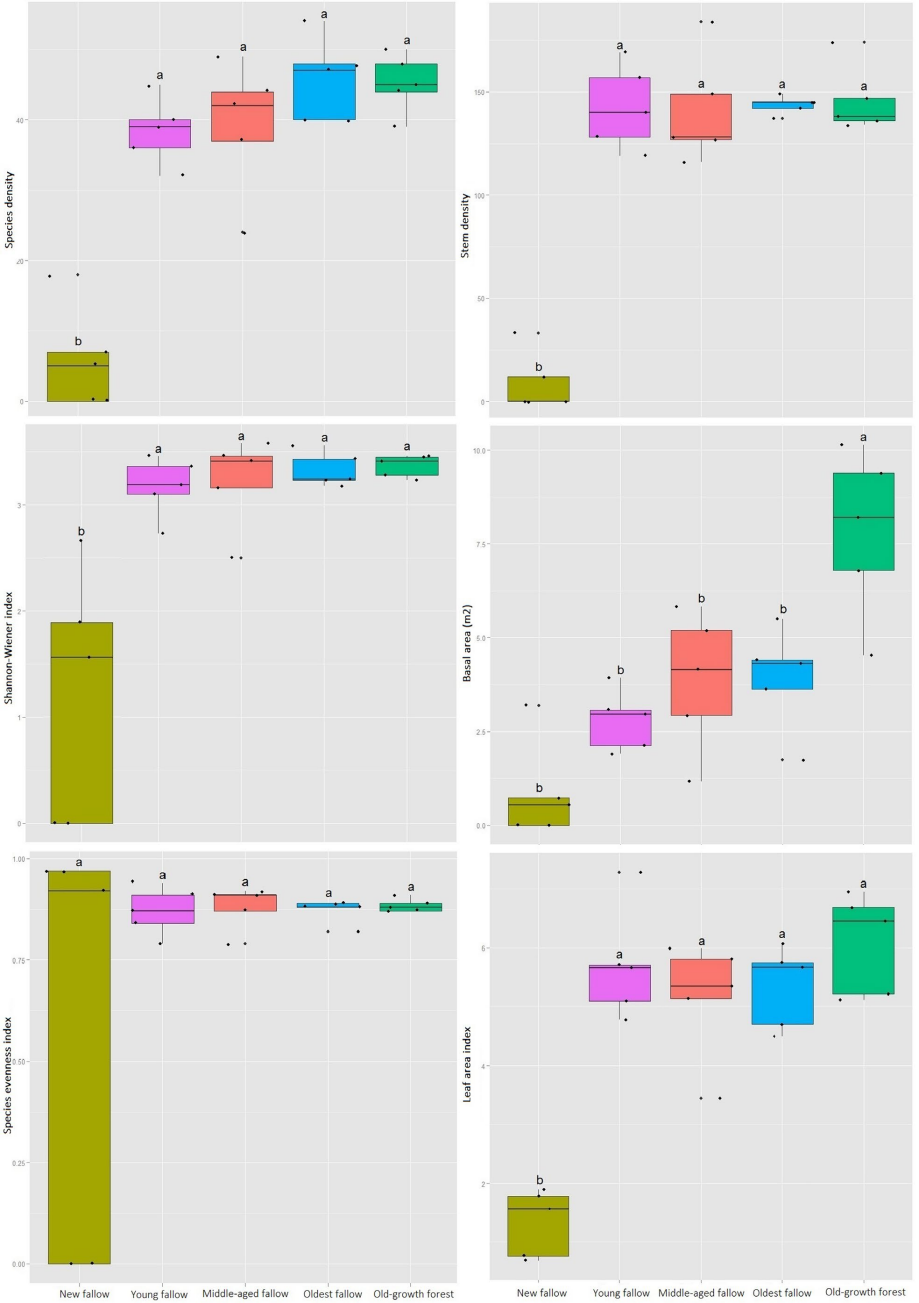
Within and between variations of tree diversity (left) and forest structure (right) indices across the sites on Leyte Island, the Philippines. Note the differences in scale on Y‐axes

Tree diversity and forest structure varied significantly across our sites. This was the case whether these attributes were measured as species density (*F*_4, 24_ = 30.79, *p* < .01), Shannon's diversity index (*F*_4, 24_ = 12.39, *p* < .01), species evenness (*F*_4, 24_ = 1.66, *p* < .05), stem density (*F*_4, 24_ = 54.1, *p* < .01), basal area (*F*_4, 24_ = 12.51, *p* < .01), or LAI (*F*_4, 24_ = 26.48, *p* < .01). Post hoc analysis using Tukey's HSD showed species density to be significantly higher (*p* < .01) in the oldest fallow secondary forest sites followed by old‐growth forest (45.2 ± 4.21) and the middle‐aged secondary forest sites (39.2 ± 9.52) (Figure 2). The Shannon's diversity index (3.37 ± 0.11), species evenness (0.88 ± 0.02), stem number (145.8 ± 16.53), tree basal area (7.81 ± 2.23), and LAI were all significantly (*p* < .001) higher in our old‐growth forest.

### 3.3 Species composition in regenerating secondary forests after shifting cultivation abandonment

Climax species had the highest IVI in the old‐growth forest and all secondary forests of different fallow age categories except in the young fallow secondary forests (Appendix 3). *Shorea polysperma* showed the highest IVI (44.34) in new fallow sites, whereas in young fallows *Lithocarpus llanosil* had the highest IVI (15.4). *Parashorea malannoan* had the highest IVI in the old‐growth forest (53.07), and in the middle‐aged (22.90), and oldest fallow sites (24.63).

**FIGURE 3:**
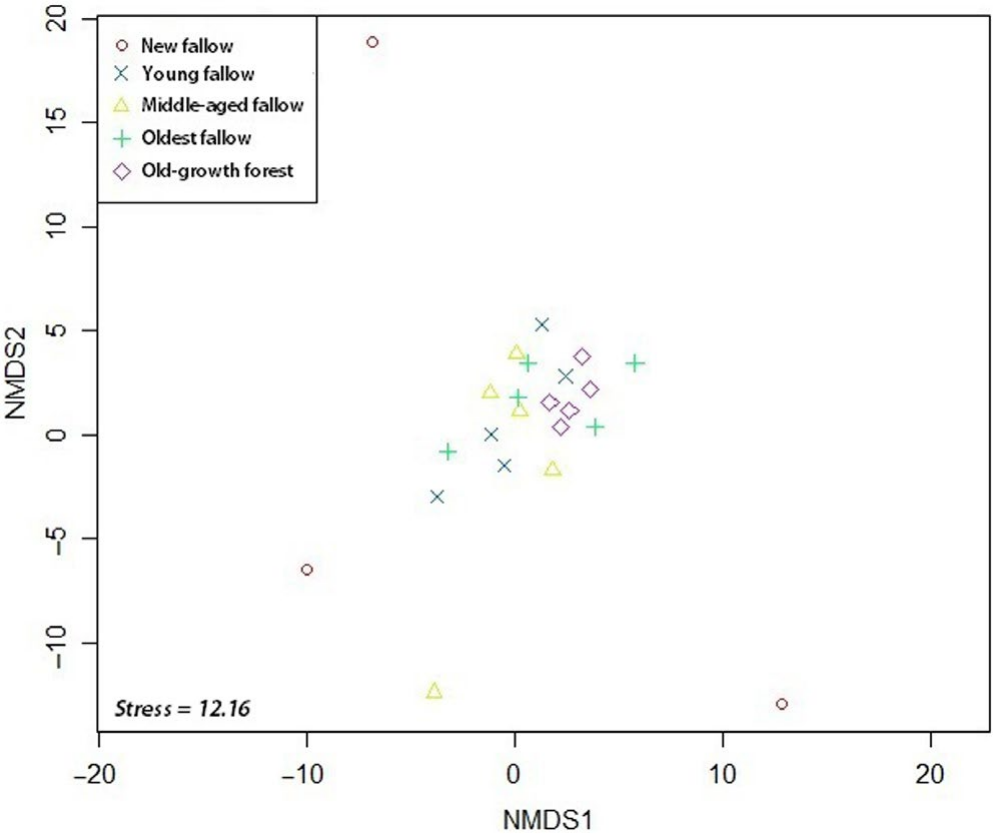
NMDS ordination of our sites using species abundance on Leyte Island, the Philippines using Bray–Curtis distance

**FIGURE 4:**
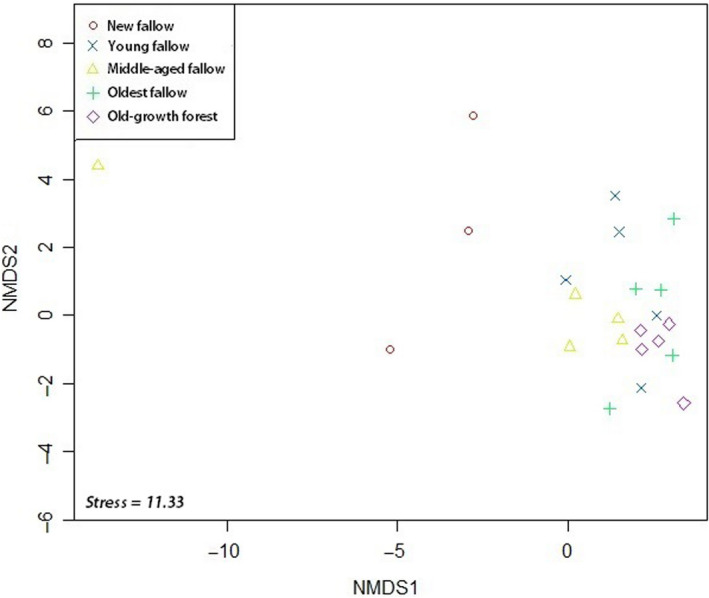
NMDS ordination of our sites using species of global conservation concern (according to IUCN Red List) on Leyte Island, the Philippines

**FIGURE 5:**
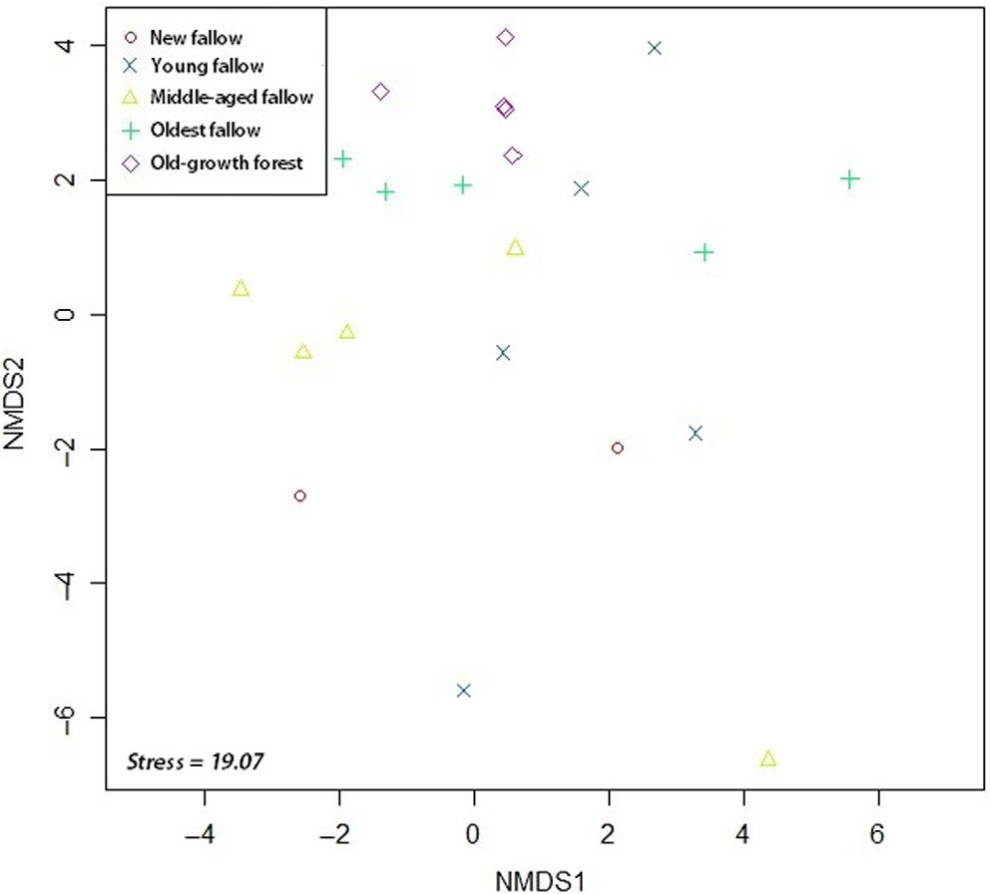
NMDS ordination of our sites using species of local conservation concern (according to DENR) on Leyte Island, the Philippines

We found a homogeneous pattern of tree species richness in our ordination. In NMDS, species abundance in secondary forests of different fallow age was clustered together with our old‐growth forest sites. There was no distinct pattern in secondary forests of older fallow age and our control forest sites (Figure 3). However, our new and young fallow secondary forest sites demonstrated different species composition as in ordination several sites were spread out. Ordination using species of global and local conservation concern also provided a similar outcome (Figures 4 and 5). PERMANOVA using site category as the main effect showed significant differences (*F*_4, 24_ = 1.88; *p* < .001) in species composition across the sites. Tukey's HSD also confirmed significantly different species composition between our old‐growth forest and the secondary forests of different fallow age categories except the oldest fallow secondary forest sites.

### 3.4 Tree diversity and forest structure recovery along a fallow age gradient

**FIGURE 6:**
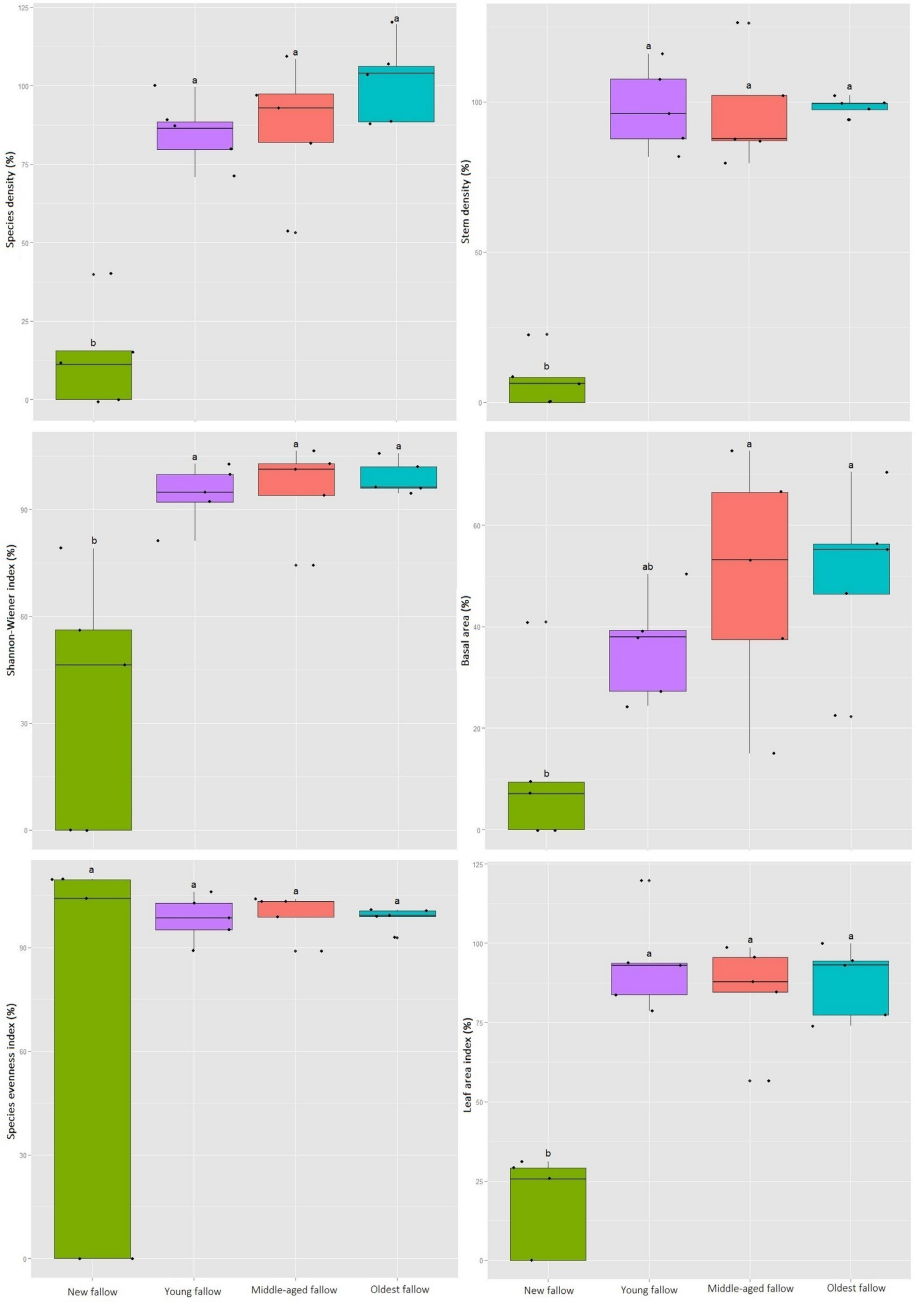
Recovery of tree diversity (left) and forest structure (right) in relation to old‐growth forest on Leyte Island, the Philippines. Note the differences in scales on *Y*‐axes

The degree of recovery of tree diversity and forest structure with respect to the old‐growth forest were significantly different across secondary forests of different fallow age categories as measured by species density (*F*_3, 19_ = 17.39; *p* < .00), Shannon's index (*F*_3, 19_ = 9.52; *p* < .001), stem density (*F*_3, 19_ = 36.06; *p* < .00), and LAI (*F*_3, 19_ = 16.28; *p* < .00). There were, however, no significant differences in the recovery of species evenness (*F*_3, 19_ = 2.43; *p* = .10) and basal area (*F*_3, 19_ = 2.34; *p* < .11) across our sites (Figure 6). Recovery of tree diversity in terms of species density (101.33 ± 13.13) and Shannon's diversity index (98.87 ± 4.78) was highest in the oldest fallow secondary forest. Recovery of forest structure parameters, including stem density (98.49 ± 3.05) and basal area (50.15 ± 17.80) was also highest in the oldest fallow sites. Our post hoc analysis using Tukey's HSD revealed a significantly lower (*p* < .05) recovery of species density, Shannon's index, stem density, and LAI in new fallow sites compared to the secondary forest sites of other fallow age categories.

### 3.5 Environmental controls on the recovery of secondary forest diversity and structure

Our multiple candidates LMM models explained the differences in species diversity and forest structure recovery in regenerating secondary forests after shifting cultivation abandonment (Table 3). For all response variables, models that included patch size explained as much variation as other more complex models that included interactions (all models within ∆AICc = 4 were considered equivalent). Other than patch size, soil organic carbon and fallow age were also found to explain variation in the recovery of different biodiversity and forest structure parameters (Table 4). Distance from old‐growth forest was not retained in any of the best supported candidate models.

**TABLE 3:** Summary of linear mixed effect models within ∆ AICc = 4 obtained using MuMin package in R (Bartoń, 2011). Where S = species density, H = Shannon's index, J = species evenness, *N* = stem density, BA = basal area, LAI = leaf area index, FA = fallow age, DIS = distance from the nearest old‐growth forest, SL = slope, PS = patch size, SOC = soil organic carbon

Response variable	Site environmental attribute					*df*	LL	AICc	∆AICc	Weight
	FA	DIS	SL	PS	SOC					
S	X			X		6	−69.93	159.50	0.0	0.36
				X		5	−72.59	160.19	0.69	0.26
				X	X	6	−70.52	160.68	1.18	0.20
	X			X	X	7	−67.85	160.9	1.4	0.18
H				X		5	−65.48	145.96	0.0	0.39
	X			X		6	−63.67	146.97	1.02	0.23
	X				X	6	−63.68	146.99	1.03	0.23
	X			X	X	7	−61.84	148.89	2.93	0.09
			X	X		6	−64.98	149.6	3.64	0.06
J				X	X	6	−49.31	118.26	0.0	0.57
				X		5	−51.9	118.8	0.55	0.43
N				X	X	6	−68.1	155.83	0.0	0.29
	X			X	X	7	−65.69	156.58	0.76	0.2
	X			X		6	−68.83	156.91	1.08	0.17
				X		5	−71.06	157.12	1.3	0.15
	X				X	6	−69.53	158.7	2.87	0.07
					X	5	−71.94	158.89	3.06	0.06
			X	X		6	−69.69	159.01	3.19	0.06
BA				X	X	6	−69.76	159.17	0.0	0.18
				X		5	−72.27	159.53	0.37	0.15
	X			X		6	−70.05	159.73	0.56	0.14
	X		X	X		7	−67.37	159.94	0.77	0.12
	X			X	X	7	−67.39	159.99	0.82	0.12
			X	X		6	−70.36	160.35	1.19	0.1
			X	X	X	7	−68.22	161.65	2.48	0.05
	X		X			6	−71.26	162.16	2.99	0.04
	X				X	6	−71.47	162.57	3.41	0.03
	X		X	X	X	8	−65.3	162.6	3.43	0.03
					X	5	−73.91	162.82	3.65	0.03
LAI				X		5	−70.67	156.35	0.0	0.35
	X			X		6	−68.72	157.07	0.72	0.25
				X	X	6	−68.72	157.08	0.74	0.24
	X			X	X	7	−66.76	158.71	2.37	0.11
			X	X		6	−70.31	160.25	3.9	0.05

Abbreviations: AICc, Akaike information criterion corrected for small sample size; *df*, degree of freedom; LL, log likelihood.

**TABLE 4:** **T**he relative importance of site environmental attributes in the final LMM. Where S = species density, H = Shannon's index, J = species evenness, N = stem density, BA = basal area, LAI = leaf area index, FA = fallow age, PS = patch size, SL = slope, DIS = distance from the nearest old‐growth forest, SOC = soil organic carbon

Site indices	Site environmental attribute^a^					Number of models
	FA	PS	DIS	SL	SOC	
S	0.54 (2)	1.0 (4)	—	—	0.38 (2)	4
H	0.32 (2)	1.0 (5)	—	0.06 (1)	0.32 (2)	5
J	—	1.0 (2)	—	—	0.57 (1)	2
N	0.44 (3)	0.87 (5)	—	0.06 (1)	0.62 (4)	7
BA	0.49 (6)	0.9 (8)	—	0.35 (5)	0.45 (6)	11
LAI	0.35 (2)	1.0 (5)	—	0.05 (1)	0.35 (2)	5

^a^ Values in the parenthesis indicates the number of models containing a respective explanatory variable.

## 4 DISCUSSION

### 4.1 Biodiversity conservation and secondary forest development after shifting cultivation abandonment

Our study has revealed rapid recovery of secondary forests regenerating after shifting cultivation abandonment in the study area and the important role of these forests in species conservation at the landscape level. Recovery of tree diversity was more rapid than forest structure. Although there was a substantial decrease in biodiversity in the first five years, our older fallow secondary forest sites exhibited similar levels of biodiversity to that of old‐growth forest in the area. Our study approach and measures of biodiversity (i.e., species density, Shannon's index, and species evenness) have been widely used to investigate the successional development of forest after disturbance cessation (see—Jakovac et al., 2015; Letcher & Chazdon, 2009; Martin, Newton, & Bullock, 2013; Norden et al., 2015; Villa, Martins, Oliveira Neto, Rodrigues, Martorano, et al., 2018; Villa, Martins, Oliveira Neto, Rodrigues, Vieira, et al., 2018). We found that Shannon's index and species evenness index were higher in old‐growth forest and that species density was higher in the oldest fallow secondary forest. Recently, abandoned new fallow sites demonstrated a very distinct pattern of species diversity compared to the secondary forest sites of much older fallow age categories in our study. A similar observation was made by Klanderud et al. (2010) in Madagascar who found shrub dominance after the abandonment of shifting cultivation was inhibiting tree diversity in the area. Young fallow areas have less diversity of adult tree species (Miller & Kauffman, 1998; Schmook, 2010), and woody species become prominent in secondary fallow forests after about ten years (Gemerden, Shu, & Olff, 2003; Klanderud et al., 2010; Raharimalala et al., 2010). Some studies suggest that secondary forest, for example, after selective logging, can exhibit greater forest structure in terms of basal area than undisturbed forest (e.g., Castro‐Luna et al., 2011; Ding et al., 2012), although the conservation value of such landscapes are not always high when considering the diversity of forest specialist or climax species (Fredericksen & Mostacedo, 2000; Mo, Zhu, Zhang, Slik, & Liu, 2011). Despite a comparable stem density in the older fallow secondary forests sites and the old‐growth forest, tree basal area was significantly higher our control old‐growth forest sites. This may be attributed to the presence of large diameter trees in the area, as also experienced by Mo et al. (2011).

Despite a relatively homogeneous species composition in our old‐growth forest sites and secondary forests of older fallow age, the new fallow sites showed a limited overlap in species composition with our control old‐growth forest. Species composition is regarded as a key aspect of the study of forest recovery after disturbance (Slik, Bernard, Beek, Breman, & Eichhorn, 2008), and several studies have reported that the similarity between undisturbed forests and regenerating secondary forests will increase with an increase in the abandonment age (see—Lawrence, 2004; Norden et al., 2009; Piotto et al., 2009; Rozendaal et al., 2019). In NMDS ordination, two of our new fallow sites were wholly spread out, which may be a consequence of the intensity of past land use represented by fallow cycles that we were not able to incorporate into our analysis. Interestingly, when considering IVI of species, some dipterocarp species (e.g., *Parashorea malaanoan*) were found to be dominant across all sites. This may be attributed to such remnant large‐canopy trees not interfering during the time of agricultural use (Häger, Otárola, Stuhlmacher, Castillo, & Arias, 2014; Sandor & Chazdon, 2014).

Recovery of species density and Shannon's index was rapid in our older fallow secondary forests sites, although we found no significant difference in the recovery of species evenness. Recovery of stem density increased gradually with the fallow age. Recovery of stand basal area was distinct across the sites and was more inclined to our older fallow secondary forests sites, which is an indication of the greater number of large and mature trees in those sites. In tropical regions, the degree to which a forest recovers after disturbance is uncertain (Ding et al., 2012), although it is known that following disturbance tropical forests can recover well in terms of tree diversity and stand structure (see—Cannon, Peart, & Leighton, 1998; Letcher & Chazdon, 2009; Rozendaal et al., 2019). Unlike Slik et al. (2008), Letcher and Chazdon (2009) and Martin et al. (2013) who found rapid recovery of forest structure parameters over diversity during secondary forest succession, we found rapid recovery of forest diversity parameters in our secondary forest sites.

### 4.2 Controls of site environmental factors in forest recovery after shifting cultivation abandonment

Patch size influences the recovery of species diversity and composition in our study sites, and patch size on its own was found to have a similar variable importance for recovery of forest diversity and structure as more complicated models that include interactions of fallow age, slope, and soil organic carbon. Echeverría, Newton, Lara, Benayas, and Comes, 2007 also found patch size as the single most important factor influencing both species composition and stand structure in terms of basal area and stem density. Both fallow age and fallow cycles influence the intensity of past forest use and their ability to recover (Lawrence, 2004; Schmook, 2010; Villa, Martins, Oliveira Neto, Rodrigues, Martorano, et al., 2018), and however, in our analysis we only included fallow age. Biodiversity recovery in tropical secondary forests may depend on the remaining forest cover (Arroyo‐Rodriguez et al., 2016; Arroyo‐Rodriguez, Pineda, Escobar, & Benitez‐Malvido, 2008), although the intensity of past land use is a stronger predictor of forest recovery than edaphic environmental variables, highlighting the importance of humans in shaping tropical forest dynamics (Castro‐Luna et al., 2011; Ding et al., 2012; Klanderud et al., 2010; Mukul, Herbohn, & Firn, 2016b).

Our study's investigation of soil parameters was limited to soil organic carbon, which is an important indicator of soil fertility. Despite this limitation, our findings agree with those of Paoli, Curran, and Slik (2008) and Poorter et al. (2016), who found soil nutrients positively influence the recovery of forest structure parameters like basal area. We, however, found no significant effect of distance from the nearest old‐growth forest on the recovery of any of the parameters investigated in our study as also reported by Piotto et al. (2019) in Brazil. Studies on secondary forest dynamics have demonstrated that diversity of woody species increases gradually with time, although the rate of recovery differs depending on the geographic location of the site and the associated environmental parameters (see—Chazdon, 2014; Ehrlen & Morris, 2015; Klanderud et al., 2010; Lawrence et al., 2010; Martin et al., 2013; Piotto et al., 2009; Poorter et al., 2016; Read & Lawrence, 2003; Sovu, Tigabu, Savadogo, Odén, & Xayvongsa, 2009; Uddin, Steinbauer, Jentsch, Mukul, & Beierkuhnlein, 2013). Previous studies (e.g. Ding et al., 2012; N'Dja & Decocq, 2008; Schmook, 2010) have reported that secondary forests may require different durations to attain the diversity and structure of an undisturbed forest after they have been abandoned. Fallow age, however, may not affect the recovery of forest diversity measures when a common species pool occurs across the sites (Sovu et al., 2009). Connectivity to primary forests increases forest regeneration by influencing natural processes like pollination and seed dispersal (Echeverría et al. (2007); Häger et al., 2014) although in our study it was not important. A declining trend in species richness with increasing distance to primary forest edges, however, may be found (Sovu et al., 2009).

### 4.3 Implications for forest and landscape restoration and conservation

Our study confirms that regenerating forests in the tropics have the ability to recover after shifting cultivation, although site factors (patch size in our case) may be important during this recovery process. Throughout the tropics, secondary forests are expanding dramatically, and in many countries, they have already exceeded the total area covered by remaining primary or old‐growth forests (Chazdon, 2014; McNamara et al., 2012). Such forests have emerged following intensive human land use, and management interventions are being increasingly considered because these emerging “novel” regrowth ecosystems commonly have a different composition and structure than the original forest type (Hobbs, Higgs, & Harris, 2009). The successful maintenance of tree diversity in such ecosystems offers important synergies for conservation, as high plant diversity is associated with higher wildlife diversity (Chazdon et al., 2009). In the case of the Philippines, secondary forest covers a large area and represents a highly dynamic ecosystem, making biodiversity conservation a daunting challenge in the country (Lasco et al., 2001; Posa et al., 2008).

We found that secondary forests regenerating after *kaingin* abandonment can also support relatively high numbers of rare and endangered species and thus can play an important role in their conservation. This is contrary to previous views that fallow landscapes are unfavorable for the maintenance forest biodiversity (see—Gemerden et al., 2003; Mo et al., 2011). The population status of rare and endemic species is highly relevant to the prioritization of conservation efforts globally (Kier et al., 2009). Tropical forest‐agriculture frontiers also provide important clues for conservation in complex, heterogeneous landscapes (Kumaraswamy & Kunte, 2013). We found that the number of endemics, climax and red‐listed species was greater in relatively older fallow sites. A number of dipterocarp species, including *Hopea philippinensis, Shorea polysperma,* and *Shorea contorta*—that are of both local and global conservation significance, were shared among secondary forests of different fallow age categories and in control old‐growth forest, indicating a high conservation value of regenerating secondary forests.

## 5 CONCLUSIONS

It is clear that forests regenerating following the abandonment of shifting cultivation have the potential to mitigate the loss of tropical forest biodiversity. It is also clear that these secondary forests have a high resilience or capacity to recover. In our study, we found that species composition in secondary forests with fallow ages of more than five years were similar to the less‐disturbed old‐growth forest, indicating a continuous accumulation of tree species after abandonment. Recovery of tree diversity was rapid compared to the forest structure, and in all cases, new fallow secondary forest showed a more divergent pattern of forest structure and diversity compared to that of our older fallow secondary forest categories. Although shifting cultivation has a negative reputation for causing the degradation and loss of tropical rainforests, little attention has been paid to studying secondary forest dynamics in complex, human‐modified landscapes. Our study indicates that novel ecosystems such as fallow secondary forests that arise after deliberate human intervention can recover rapidly after disturbance and can act as a low‐cost refuge for biodiversity conservation. Such forests can also serve as a low‐cost ecosystem restoration measure in tropical developing countries where shifting cultivation is common.

## CONFLICT OF INTEREST

The authors declare no conflict of interest.

## AUTHOR CONTRIBUTION

**Sharif A. Mukul:** Conceptualization (equal); Data curation (equal); Formal analysis (equal); Investigation (equal); Methodology (equal). **John Herbohn:** Conceptualization (equal); Funding acquisition (equal); Methodology (equal); Project administration (equal). **Jennifer Firn:** Conceptualization (equal); Formal analysis (equal); Investigation (equal); Methodology (equal); Project administration (equal).

## Data Availability

Additional data related to this study is available at Dryad Data Repository (https://doi.org/10.5061/dryad.nvx0k6dph).

## APPENDIX 1

Pearson correlation between environmental attributes of our sites on Leyte Island, the Philippines.

**Table:**

	Fallow age	Elevation	Slope	Patch size	Distance	SOC
Fallow age	1					
Elevation	−0.09	1				
Slope	0.25	0.52*	1			
Patch size	0.07	−0.01	−0.16	1		
Distance	−0.35	−0.12	0.07	−0.09	1	
SOC	0.05	0.53*	0.43	−0.325	−0.005	1

^*^Correlations significant at *p* < 0.05 level.

## APPENDIX 2

List of tree species recorded from our sites on Leyte Island, the Phillipines.

**Table:**

Family	Botanical name^a^	Local name	Abundance					Origin^b^	Conservation status		Successional guild^e^
			New fallow	Young fallow	Middle‐aged fallow	Oldest fallow	Old‐growth forest		IUCN^c^	DENR^d^	
Alangiaceae	*Alangium javanicum* (Bl.) Wang	Putian	—	4	3	3	5	Native	LR	NA	Secondary
Anacardiaceae	*Dracontomelon dao* (Blanco) Merr. & Rolfe	Dao	—	2	3	1	2	Native	NA	V	Secondary
	*Dracontomelon edule* (Blanco) Skeels.	Lamio	—		2	1	1	Native	NA	V	Secondary
Annonaceae	*Cananga odorata* (Lam.) Hook. f. & Thomson	Ilang‐ilang	—	1	—	—	—	Native	NA	NA	Secondary
	*Polyalthia oblongifolia* Burck	Lapnisan	—	6	2	6	1	Native	NA	NA	Secondary
	*Polyalthia flava*	Yellow lanutan	—	7	4	1	8	Endemic	NA	NA	Secondary
Apocynaceae	*Alstonia macrophylla* G. Don	Batino	—	5	4	5	—	Native	LR	NA	Pioneer
	*Alstonia parvifolia* Merr.	Batino‐liitan	—	—	3	—	—	Endemic	NA	NA	Pioneer
	*Wrightia pubescens* R.Br.	Lanete	1	9	13	9	2	Native	NA	NA	Pioneer
	*Kibatalia gitingensis* (Elmer) Woodson	Laneteng‐gubat	—	1	1	17	8	Endemic	V	CE	Secondary
Araliaceae	*Arthrophyllum cenabrei* Merr.	Bingliu	—	—	9	—	—	Endemic	NA	NA	Pioneer
	*Polyscias nodosa* (Blume) Seem.	Malapapaya	2	51	83	97	14	Native	NA	NA	Pioneer
Arecaceae	*Areca cathecu* L	Bunga	—	—	5	8	—	Native	NA	NA	Secondary
	*Cocos nucifera* L.	Coconut	—	—	—	5	—	Native	NA	NA	Pioneer
	*Caryota cumingii* Lodd. ex Mart.	Pugahan	—	43	25	26	83	Native	NA	NA	Pioneer
	*Heterospathe elata* Scheff.	Sagisi	—	6	6	2	15	Native	NA	NA	Pioneer
Bignoniaceae	*Radermachera pinnate (Blanco) Seem.*	Banai‐Banai	2	28	24	11	2	Native	NA	NA	Secondary
Burseraceae	*Canarium hirsutum*	Milipili	—	—	2	6	1	Native	NA	NA	Secondary
	*Canarium calophyllum* Perkins.	Pagsahingin‐bulog	1	11	10	8	12	Native	NA	NA	Secondary
	*Canarium luzonicum* (Blume) A.Gray	Piling‐liitan	—	2	3	6	28	Endemic	V	NA	Pioneer
Calophyllaceae	*Calophyllum blancoi* Planch. & Triana	Bitanghol	—	7	7	22	9	Native	NA	NA	Secondary
	*Calophyllum lancifolium* Elmer.	Bitanghol‐sibat	2	3	—	2	—	Native	NA	NA	Secondary
Cannabaceae	*Trema orientalis* (L.) Bl.	Anabiong	1	—	—	—	2	Native	NA	NA	Pioneer
	*Celtis philippensis* Blanco	Malaikmo	—	—	—	1	4	Native	NA	NA	Secondary
Casuarinaceae	*Casuarina equisetifolia* L.	Agoho	1	—	1	—	—	Exotic	NA	NA	Pioneer
	*Gymnostoma rumphianum* (Miq.) L.A.S. Johnson	Mountain agoho	1	—	—	1	—	Native	NA	NA	Secondary
Combretaceae	*Terminalia microcarpa* Decne.	Kalumpit	—	—	2	—	—	Native	NA	NA	Secondary
Cycadaceae	*Cycas circinalis* L.	Pitogo	—	—	1	—	—	Native	E	NA	Pioneer
Datiscaceae	*Octomeles sumatrana* Miq.	Binuang	—	—	—	1	—	Native	LR	NA	Secondary
Dilleniaceae	*Dillenia indica* L.	Handapara	—	2	—	4	—	Native	NA	NA	Secondary
	*Dillenia philippinensis* Rolfe	Katmon	—	2	6	5	6	Endemic	V	NA	Secondary
Dipterocarpaceae	*Shorea almon* Foxw.	Almon	—	2	—	3		Endemic	CE	V	Climax
	*Parashorea malaanonan* (Blanco) Merr.	Bagtikan	4	11	19	32	61	Native	NA	NA	Climax
	*Dipterocarpus eurynchus* Miq.	Basilanapitong	—	—	1	—	—	Endemic	CE	E	Secondary
	*Hopea philippinensis* Dyer	Gisok‐gisok	—	7	3	2	20	Endemic	CE	CE	Pioneer
	*Shorea guiso* (Blanco) Blume	Guijo	—	15	8	4	31	Native	CE	NA	Pioneer
	*Shorea palosapis* (Blanco) Merr.	Mayapis	—	3	9	5	12	Endemic	CE	NA	Climax
	*Anisoptera thurifera*	Palosapis	—	—	—	1	—	Native	NA	NA	Secondary
	*Shorea polysperma* (Blanco) Merr.	Tangile	2	6	8	14	5	Endemic	CE	V	Climax
	*Shorea contorta* Vidal	White lauan	2	5	16	6	4	Endemic	CE	V	Climax
	*Hopea malibato*	Yakal‐kaliot	—	—	—	1	—	Native	CE	CE	Climax
Ebenaceae	*Diospyros pilosanthera* Blanco	Bolong‐eta	—	1	4	9	3	Native	NA	E	Secondary
	*Diospyros blancoi* A.DC.	Kamagong	—	1	3	—	1	Native	V	CE	Climax
Euphorbiaceae	*Neotrewia cumingii* (Müll.Arg.) Pax & K.Hoffm.	Apanang	—	3	3	2	4	Native	NA	NA	Pioneer
	*Mallotus philippensis* (Lam.) Müll.Arg.	Banato	—	2	—	—	—	Native	NA	NA	Secondary
	*Macaranga tanarius* (L.) Müll.Arg.	Binunga	—	5	4	2	7	Native	NA	NA	Pioneer
	*Macaranga bicolor* Muell. ‐Arg.	Hamindang	—	9	1	8	5	Endemic	V	NA	Pioneer
	*Mallotus ricinoides* (Pers.) Müll.Arg.	Hinlaumo	1	5	12	5	—	Native	NA	NA	Pioneer
Fabaceae	*Ormosia calavensis* Blanco	Bahai	—	34	3	19	2	Endemic	NA	NA	Climax
	*Albizia falcataria* (L.) Fosberg.	Falcata	—	5		6	—	Exotic	NA	NA	Secondary
	*Leucaena leucaephala* (Lam.) de Wit.	Ipil‐ipil	—	—	7	3	—	Exotic	NA	NA	Pioneer
	*Pterocarpus indicus* Willd.	Narra	8	—	—	—	—	Native	V	NA	Secondary
	*Albizia saponaria*(Lour.) Blume ex Miq.	Salingkugi	—	4	—	3	8	Native	NA	NA	Secondary
	*Senna siamea* (Lam.) Irwin et Barneby	Thailand shower	—	—	2	1	—	Native	NA	NA	Secondary
Fagaceae	*Lithocarpus llanosii* (A.DC.) Rehder	Ulaian	2	17	28	27	16	Native	NA	NA	Pioneer
Hypericaceae	*Cratoxylum celebicum* Bl.	Paguriagon	—	4	2	11	4	Native	NA	NA	Secondary
Lamiaceae	*Vitex quinata* (Lour.) F. N. Williams	Kalipapa	—	—	1	—	10	Native	NA	NA	Pioneer
	*Vitex turczaninowii* (Turcz.) Merr.	Lingo‐lingo	—	—	—	—	1	Endemic	NA	NA	Secondary
	*Premna cumingiana* Schauer	Magilik	1	—	—	—	2	Native	NA	NA	Secondary
	*Tectona grandis* L. f.	Teak	—	—	—	1	—	Exotic	NA	NA	Pioneer
	*Callicarpa elegans* Hayek	Tigau‐ganda	—	—	—	2	—	Endemic	NA	NA	Pioneer
Lauraceae	*Cinnamomum cebuense* Kostermans	Kaningag	—	2	2	2	—	Endemic	CE	NA	Pioneer
	*Litsea perrottetii* (Bl.) Villar.	Marang	‐	5	2	8	4	Native	NA	NA	Secondary
	*Neolitsea vidalii* Merr.	Puso‐puso	—		7	3	6	Endemic	V	NA	Secondary
Lecythidaceae	*Barringtonia racemosa* Spreng.	Putat	—	3	4	6	11	Native	NA	NA	Pioneer
	*Petersianthus quadrialatus* (Merr.) Merr.	Toog	—	—	—	1	8	Endemic	NA	NA	Pioneer
Malvaceae	*Diplodiscus paniculatus* Turcz.	Balobo	—	9	5	7	5	Endemic	V	NA	Secondary
	*Pterospermum obliquum* Blanco	Kulatingan	—	—	—	1	2	Endemic	NA	NA	Pioneer
Melastomataceae	*Astronia cumingiana* S.Vidal	Badling	—	4	8	7	3	Native	CE	NA	Pioneer
Meliaceae	*Dysoxylum decandrum* Merrill.	Igyo	—	2	3	2	6	Native	NA	NA	Pioneer
	*Toona philippinensis* Elmer.	Lanigpa	—	1	3	5	4	Native	NA	NA	Pioneer
	*Dysoxylum cumingianum* C. DC.	Tara‐tara	—	4	6	13	10	Native	NA	NA	Pioneer
Moraceae	*Artocarpus blancoi* (Elmer) Merr.	Antipolo	1	6	5	4	7	Endemic	V	NA	Pioneer
	*Artocarpus ovatus* Blanco	Anubing	—	—	—	2	—	Endemic	NA	NA	Pioneer
	*Ficus irisana* Elmer.	Aplas	—	5	9	7	1	Native	NA	NA	Pioneer
	*Ficus balete* Merr.	Balete	1	9	10	—	4	Native	NA	NA	Pioneer
	*Ficus gul* K. Schum. & Lauterb.	Butli	3	41	19	32	18	Native	NA	NA	Secondary
	*Ficus minahassae* (Teijsm. & De Vriese) Miq.	Hagimit	4	19	15	4	4	Native	NA	NA	Secondary
	*Ficus septica* Burm. f.	Hauili	2	3	18		—	Native	NA	NA	Secondary
	*Ficus ulmifolia* Lam.	Is‐is	—	3	1	2	1	Endemic	V	NA	Pioneer
	*Ficus callosa* Willd.	Kalukoi	—	—	2	1	—	Native	NA	NA	Pioneer
	*Ficus magnoliifolia* Blume	Kanapai	—	6	2	1	—	Native	NA	NA	Secondary
	*Ficus odorata* (Blanco) Merr.	Pakiling	—	1	1	5	—	Endemic	NA	NA	Climax
	*Ficus vrieseana* Miq.	Tagitig	—	—	—	1	1	Native	NA	NA	Secondary
	*Ficus nota* Merr.	Tibig	1	21	17	2	7	Native	NA	NA	Pioneer
	*Ficus ampelas* Burm. f	Upling‐gubat		12	1	—	6	Native	NA	NA	Pioneer
Myricaceae	*Myrica javanica* Reinw. ex Bl.	Hindang	1	—	—	—	3	Native	NA	NA	Secondary
Myristicaceae	*Knema mindanaensis* (Warb.) comb. nov.	Bunod	—	1	3	5	—	Endemic	NA	NA	Secondary
	*Parartocarpus venenosus* (Zoll. & Morr.) Becc. ssp. papuanus (Becc.) Jarr.	Malanangka	1	2	2	2	4	Native	NA	NA	Pioneer
	*Horsfieldia costulata* (Miq.) Warb.	Yabnob	—	33	36	26	38	Native	NA	NA	Pioneer
Myrtaceae	*Syzygium surigaense* (Merr.) Merr.	Kagagko	—	—	—	2	3	Endemic	NA	NA	Secondary
	*Syzygium crassilimbum* (Merr.) Merr.	Kaitatanag	—	1	1	2	—	Endemic	NA	NA	Secondary
	*Syzygium gigantifolium* (Merr.) Merr.	Malatalisai	—	4		4	4	Endemic	NA	NA	Secondary
	*Syzygium hutchinsonii* (C.B.Robinson) Merr.	Malatambis	1	10	11	12	5	Endemic	NA	NA	Secondary
	*Xanthostemon verdugonianus* Naves	Mangkono	—	—	—	2	2	Endemic	V	NA	Pioneer
Olacaceae	*Strombosia philippinensis* (Baill.) Rolfe	Tamayuan	—	12	14	14	16	Endemic	NA	NA	Secondary
Pandanaceae	*Pandanus radicans* Blanco	Ulangong‐ugatan	—	—	—	2	—	Endemic	NA	NA	Pioneer
Phyllanthaceae	*Securinega fIexuosa* (Muell. ‐Arg.)	Anislag	—	6	4	6	—	Endemic	NA	NA	Pioneer
	*Antidesma ghaesembilla* Gaertn.	Binayuyu	—	13	—	3	3	Native	NA	NA	Climax
	*Glochidion camiguinense* Merr.	Bunot‐Bunot	—	8	3	10	2	Endemic	NA	NA	Pioneer
	*Glochidion album* (Blanco) Boerl.	Malabagang	—	1	1	2	3	Native	NA	NA	Pioneer
	*Breynia rhamnoides* Müll.Arg.	Matang‐hipon	—	3	2	2	—	Native	NA	NA	Pioneer
	*Cleistanthus venosus* C.B. Rob.	Sarimisim	—	2		1	—	Endemic	NA	NA	Secondary
	*Bridelia penangiana* Hook.f.Bridelia insulana Hance	Subiang	—	3	1	1	8	Native	N	N	Secondar
	*Bischofia javanica* Blume	Tuai	—	24	20	26	58	Native	NA	NA	Secondary
Piperaceae	*Piper anduncum* L.	Spiked pepper	3	—	3	1	5	Native	NA	NA	Pioneer
Primulaceae	*Ardisia pyramidalis* (Cav.) Pers. ex A. DC.	Aunasin	—	8	6	4	12	Native	NA	NA	Secondary
Rhizophoraceae	*Carallia brachiata* (Lour.) Merr.	Bakauan‐gubat	—	4	2	1	1	Native	NA	NA	Secondary
Rubiaceae	*Neonauclea formicaria* (Elmer) Merr.	Hambabalud	—	6	7	7	2	Endemic	NA	NA	Pioneer
	*Mussaenda philippica* A.Rich.	Kahoi‐dalaga	1	1	—	—	—	Native	NA	E	Secondary
	*Neonauclea bartlingii* (DC.) Merr.	Lisak	—	1	6	4	5	Endemic	NA	NA	Secondary
	*Canthium fenicis* (Merr.) Merr.	Mapugahan	—	2	—	4	—	Endemic	NA	NA	Pioneer
	*Canthium monstrosum* (A. Rich.) Merr.	Tadiang‐anuang	—	4	1	1	—	Native	NA	NA	Pioneer
Rutaceae	*Melicope triphylla* (Lam.) Merr.	Matang‐arau	—	4	4	3	1	Endemic	NA	NA	Pioneer
Sapindaceae	*Nephelium lappaceum*	Rambutan	—	—	1	—	—	Native	LR	NA	Pioneer
Sapotaceae	*Planchonella nitida* (Blume) Dubard.	Duklitan	—	—	1	9	—	Exotic	NA	NA	Secondary
	*Palaquium luzoniense* (Fern.‐Vill.) Vidal	Nato	—	11	2	8	16	Endemic	V	V	Secondary
Sterculiaceae	*Pterospermum diversifolium* Bl.	Bayok	—	—	4	1	1	Native	NA	NA	Pioneer
Tiliaceae	*Trichospermum involucratum* (Merr.) Elmer	Langosig	—	—	1	—	—	Endemic	NA	NA	Pioneer
Urticaceae	*Leucosyke capitellata* (Pair.) Wedd.	Alagasi	3	46	60	5	7	Native	NA	NA	Pioneer
	*Pipturus arborescens* (Link) C. B. Rob.	Dalunot	—	—	1	8	—	Native	NA	NA	Pioneer
	*Dendrocnide stimulans* (L. f) Chew	Lingaton	—	—	—	1	—	Native	NA	NA	Pioneer
Verbenaceae	*Premna odorata* Blanco	Alagau	1	—	2	—	—	Native	NA	NA	Secondary
	*Premna stellate* Merr.	Manaba	—	—	1	—	—	Native	NA	NA	Secondary
Vitaceae	*Leea aculeata* Bl.	Amamali	—	1	3	—	5	Native	NA	NA	Pioneer
Unknown		Anungo	—	8	2	4	18		—	—	Secondary
		Nandamai	—	—	—	1	—		—	—	Pioneer
		Pandukaki	—	—	—	1	—		—	—	Pioneer
		Pegonngon	—	—	—	1	—		—	—	Secondary
		Poelig	—	13	1	1	—		—		Pioneer
		Siyao	1	—	—	—	—		—		Pioneer

^a^Whenever possible species name was followed as per Species 2000 & ITIS catalogue of Life, Annual Checklist (available online at: www.catalogueoflife.org/annual‐checklist/).

^b^Where Endemic ‐ refers to a species found only in the Philippines, Native – refers to a species naturally occurring in the Philippines and Exotic – refers to a species that has been introduced in the Philippines.

^c^As per IUCN Red List of Threatened Species (available online at: http://www.iucnredlist.org), where: CE or Critically endangered – refers to a species or subspecies facing extremely high risk of extinction in the wild in the immediate future. Endangered ‐ refers to a species or subspecies that is not critically endangered but whose survival in the wild is unlikely if the causal factors continue operating. V or Vulnerable ‐ refers to a species or subspecies that is not critically endangered nor endangered but is under threat from adverse factors throughout its range and is likely to move to the endangered category in the future. LR or Lower risk – refers to a species that has been evaluated, but does not satisfy the criteria for any of the categories Critically Endangered, Endangered or Vulnerable. NA or Not available/Not assessed – refers to a species that has not yet been assessed against the criteria.

^d^As per DENR Adminsitrative Order No. 2007.01, where: CE or Critically endangered ‐ refers to a species or subspecies facing extremely high risk of extinction in the wild in the immediate future. This shall include varieties, I formae or other infraspecific categories; E or Endangered ‐ refers to a species or subspecies that is not critically endangered but whose survival in the wild is unlikely if the causal factors continue operating. This shall include varieties, formag or other infraspecific categories; V or Vulnerable ‐ refers to a species or subspecies that is not critically endangered nor endangered but is under threat from adverse factors throughout its range and is likely to move to the endangered category in the future. This shall include varieties, formae or other infraspecific categories;

^e^Based on experts opinion from the Philippines.

## APPENDIX 3

Top ten species with highest importance value in sites of different fallow categories and in old‐growth forest on Leyte Island, the Philippines.

**Table:**

Site category	Species	Relative density	Relative frequency	Relative dominance	IVI
New fallow	*Shorea polysperma*	3.71	3.33	37.30	44.34
	*Parashorea malaanonan**	7.41	3.33	23.30	34.04
	*Pterocarpus indicus*	14.82	6.67	8.47	29.95
	*Calophyllum lancifolium*	3.71	3.33	7.34	14.37
	*Siyaoǂ*	1.85	3.33	9.12	14.30
	*Polyscias nodosa***	3.71	6.67	0.65	11.02
	*Ficus minahassae*	7.41	3.33	0.23	10.97
	*Lithocarpus llanosii***	3.71	3.33	3.17	10.20
	*Shorea contorta*	3.71	3.33	2.85	9.88
	*Piper anduncum*	5.56	3.33	0.16	9.05
Young fallow	*Lithocarpus llanosii***	2.39	1.56	11.39	15.34
	*Polyscias nodosa***	7.15	2.08	3.90	13.13
	*Caryota cumingii****	6.03	1.56	4.30	11.89
	*Ficus gul*	5.75	2.61	3.23	11.58
	*Radermachera pinnate*	3.93	2.08	4.50	10.51
	*Leucosyke capitellata*	6.45	1.56	2.12	10.13
	*Ormosia calavensis*	4.77	1.56	3.29	9.62
	*Parashorea malaanonan**	1.54	2.61	4.85	8.10
	*Horsfieldia costulata***	4.63	2.08	1.80	8.51
	*Bischofia javanica***	3.37	2.08	2.93	8.38
Middle‐aged fallow	*Parashorea malaanonan**	2.70	2.04	18.16	22.90
	*Polyscias nodosa***	11.79	2.55	4.33	18.67
	*Shorea contorta*	2.27	2.04	9.47	13.78
	*Leucosyke capitellata*	8.52	2.55	1.78	12.85
	*Lithocarpus llanosii***	3.98	2.04	6.43	12.45
	*Bischofia javanica***	2.84	1.02	6.34	10.20
	*Horsfieldia costulata***	5.12	2.04	2.19	9.34
	*Ficus balete*	1.42	1.02	6.68	9.12
	*Ficus minahassae*	2.13	1.53	5.17	8.83
	*Radermachera pinnate*	3.41	2.04	2.30	7.75
Oldest fallow	*Parashorea malaanonan**	4.46	2.18	17.99	24.63
	*Polyscias nodosa***	13.51	2.18	5.80	21.50
	*Shorea polysperma*	1.95	1.31	10.03	13.29
	*Calophyllum blancoi*	3.07	2.18	7.97	13.22
	*Lithocarpus llanosii***	3.76	1.75	5.74	11.25
	*Bischofia javanica***	3.62	1.75	4.47	9.84
	*Ficus gul*	4.46	1.75	1.43	7.63
	*Horsfieldia costulata***	3.62	2.18	1.51	7.31
	*Caryota cumingii ****	3.62	1.75	1.42	6.78
	*Ormosia calavensis*	2.65	1.75	1.67	6.07
Old‐growth forest	*Parashorea malaanonan**	8.37	2.21	42.49	53.07
	*Bischofia javanica***	7.96	2.21	5.96	16.13
	*Caryota cumingii****	11.39	2.21	1.50	15.10
	*Shorea guiso*	4.25	2.21	2.85	9.32
	*Horsfieldia costulata***	5.21	2.21	1.74	9.16
	*Ficus balete*	0.55	0.89	5.83	7.27
	*Petersianthus quadrialatus*	1.10	1.33	4.61	7.04
	*Calophyllum blancoi*	1.24	1.77	3.99	7.00
	*Canarium luzonicum*	3.84	2.21	0.79	6.85
	*Palaquium luzoniense*	2.20	2.21	2.19	6.60

Where: * ‐ species common across all site categories, ** ‐ species common across at least four site categories, and ***species common across at least three site categories, *ǂ* ‐ local name
